# P-715. Trends in Mycoplasma genitalium Resistance from 2019 to 2024 in Tokyo, Japan

**DOI:** 10.1093/ofid/ofaf695.927

**Published:** 2026-01-11

**Authors:** Naokatsu Ando, Daisuke Mizushima, Misao Takano, Takahiro Aoki, Ryo Kuwata, Akira Kawashima, Hiroaki Kubota, Kai Kobayashi, Morika Mitobe, Miyake Hirofumi, Kenji Sadamasu, Hiroyuki Gatanaga

**Affiliations:** National Center for Global Health and Medicine, Japan Institute for Health Security, Shinjuku-ku, Tokyo, Japan; National Center for Global Health and Medicine, Japan Institute for Health Security, Shinjuku-ku, Tokyo, Japan; National Center for Global Health and Medicine, Japan Institute for Health Security, Shinjuku-ku, Tokyo, Japan; National Center for Global Health and Medicine, Japan Institute for Health Security, Shinjuku-ku, Tokyo, Japan; National Center for Global Health and Medicine, Japan Institute for Health Security, Shinjuku-ku, Tokyo, Japan; National Center for Global Health and Medicine, Japan Institute for Health Security, Shinjuku-ku, Tokyo, Japan; Tokyo Metropolitan Institute of Public Health, Shinjuku-ku, Tokyo, Japan; Tokyo Metropolitan Institute of Public Health, Shinjuku-ku, Tokyo, Japan; Tokyo Metropolitan Institute of Public Health, Shinjuku-ku, Tokyo, Japan; Tokyo Metropolitan Institute of Public Health, Shinjuku-ku, Tokyo, Japan; Tokyo Metropolitan Institute of Public Health, Shinjuku-ku, Tokyo, Japan; National Center for Global Health and Medicine, Shinjuku-ku, Tokyo, Japan

## Abstract

**Background:**

*Mycoplasma genitalium* (MG) is a major cause of urethritis in men and cervicitis or pelvic inflammatory disease in women, and the infection has been linked to adverse reproductive outcomes. We examined trends in macrolide- and quinolone-resistance mutations in MG isolates in Tokyo.

**Methods:**

Between 1 January 2019 and 31 December 2024, we prospectively enrolled patients with MG-positive urine or anal-swab specimens at the National Center for Global Health and Medicine. Sanger sequencing detected macrolide-resistance mutations (MRMs) in domain V of 23S rRNA and quinolone-resistance mutations in the quinolone-resistance-determining regions of *parC* and *gyrA*. Temporal trends were assessed with χ² or Fisher’s exact tests, and linear change was evaluated with a Cochran–Armitage trend test. Results for an early period (2019–2020) were compared with those for a later period (2021–2024).

**Results:**

Among 216 MG infections (165 anal, 51 urine), sequencing succeeded for 168 isolates at 23S rRNA, 154 at *parC*, and 149 at *gyrA* (success rates 77.8 %, 71.3 %, and 69.0 %, respectively). MRMs occurred in 151/168 isolates (89.9 %): A2071T in 44.0 %, A2072G in 31.0 %, and A2071G in 13.1 %. MRM prevalence did not change significantly over time (p = 0.700). *ParC* mutations were present in 123/154 isolates (79.9 %) and increased from 56 % (14/25) in 2019 to 100 % (8/8) in 2024 (Cochran–Armitage p = 0.006); however, the early- versus late-period comparison showed no significant difference (71.2 % vs 83.3 %, p = 0.078). S83I (G248T) was the most frequent *parC* mutation, found in 74.7 % (115/154) of sequenced isolates, and its proportion remained stable (p = 0.133). Mutations in *gyrA* were detected in 36/149 isolates (24.2 %), mainly M95I (17.4 %); their frequency was unchanged over the study period (31 % in 2019 vs 25 % in 2024, p = 0.821). Resistance rates did not differ significantly between urine and anal samples.

**Conclusion:**

MG strains circulating in Tokyo show persistently high macrolide resistance and an upward trend in *parC* mutation, whereas *gyrA* mutations remain comparatively stable. These findings highlight the need for sustained molecular surveillance, rigorous antimicrobial stewardship, and the development of novel therapeutic options for MG infection.

**Disclosures:**

All Authors: No reported disclosures

